# Supplementary material for: Vitamin D Modified DSS-Induced Colitis in Mice via STING Signaling Pathway
Source: Biology (Basel). 2025 Jun 18;14(6):715. doi: 10.3390/biology14060715 (PMC12190092; doi:10.3390/biology14060715)
Supplement: Supplementary file 1 [file biology-14-00715-s001.zip › Supplementary Materials S1: 16s-report of gut microbiota/Groups/betadiv/pcoa_unweighted_unifrac_analysis2/Pcoa_unifrac13_boxplot.pdf]

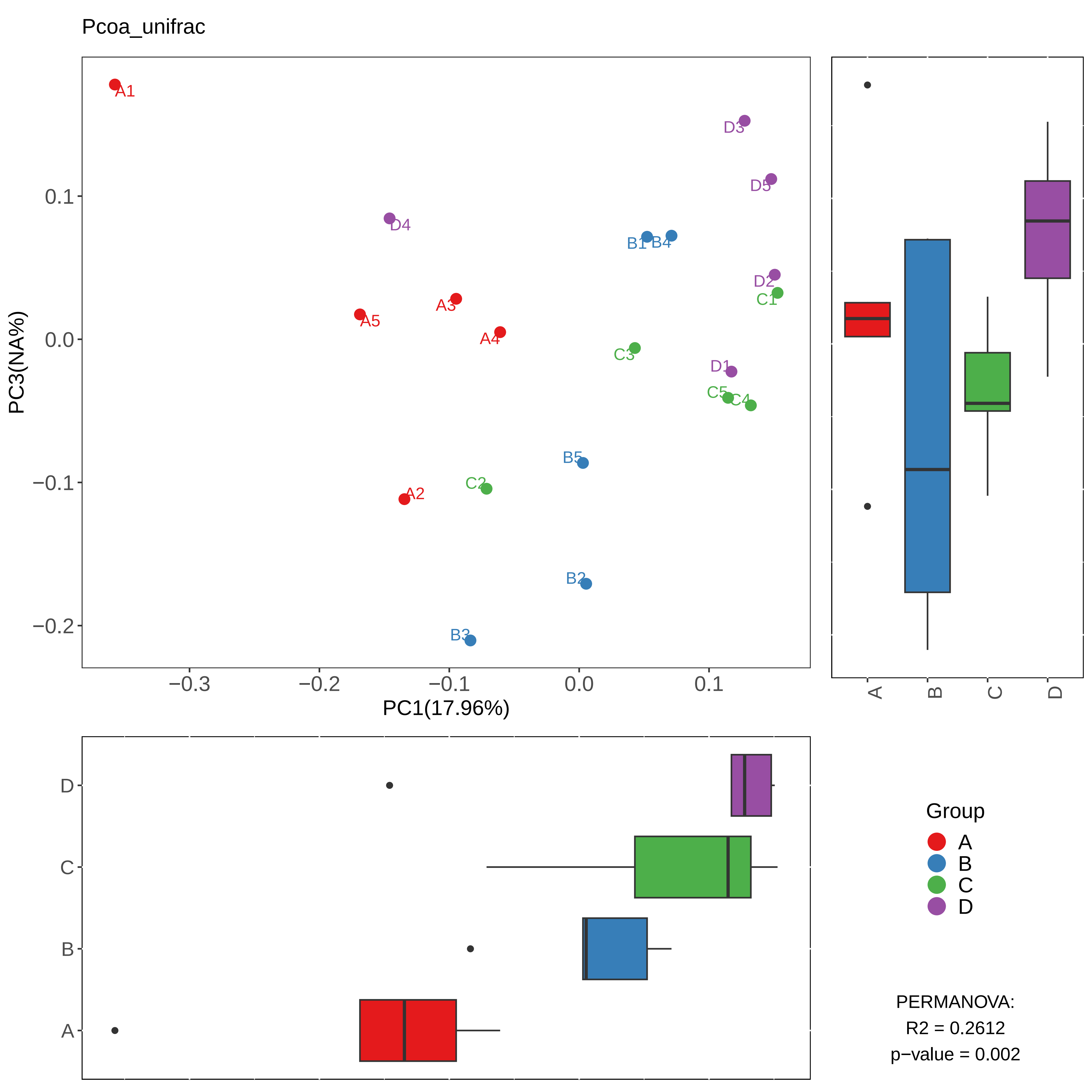
Pcoa_unifrac
A1
A2
A3
A4
A5
B1
B2
B3
B4
B5
C1
C2
C3
C4
C5
D1
D2
D3
D4
D5
0.1
0.0
−0.1
−0.2
−0.3
PC1(17.96%)
PC3(NA%)
A
B
C
D
Group
PERMANOVA:
R2 = 0.2612
p−value = 0.002
